# Prognostic value of creatine kinase (CK)-MB to total-CK ratio in colorectal cancer patients after curative resection

**DOI:** 10.1186/s12885-024-12307-5

**Published:** 2024-04-29

**Authors:** Lubei Rao, Pingyao Xu, Guiji Zhang, Ruiling Zu, Yajun Luo, Kaijiong Zhang, Ying Yang, Dongsheng Wang, Shuya He, Huaichao Luo, Bo Ye

**Affiliations:** 1 Department of Clinical Laboratory, Sichuan Clinical Research Center for Cancer, Sichuan Cancer Hospital & Institute, Sichuan Cancer Center, Affiliated Cancer Hospital of University of Electronic Science and Technology of China, No. 55, Section 4, Renmin South Road, Wuhou District, Chengdu City, Sichuan Province China; 2 Department of Gastrointestinal Surgery, Sichuan Cancer Hospital and Institute, Chengdu, Sichuan China; 3 Department of Information, Sichuan Cancer Hospital and Institute, Sichuan Cancer Center, School of Medicine, University of Electronic Science and Technology of China, Chengdu, China

**Keywords:** Colorectal cancer, Prognosis, Biomarkers, Creatine Kinase, Hepatic metastasis

## Abstract

**Objectives:**

This study aimed to evaluate the prognostic significance of postoperative Creatine Kinase type M and B (CK-MB) to total Creatine Kinase (CK) ratio (CK-MB/CK) in colorectal cancer (CRC) patients after radical resection.

**Methods:**

This was a single-center retrospective cohort analysis. Subjects were stage I-III CRC patients hospitalized in Sichuan Cancer Hospital from January 2017 to May 2021. Patients were divided into abnormal group and normal group according to whether the CK-MB/CK ratio was abnormal after surgery. Through a comparative analysis of clinical data, laboratory test results, and prognosis differences between the two groups, we aimed to uncover the potential relationship between abnormal CK-MB > CK results and CRC patients. To gauge the impact of CK-MB/CK on overall survival (OS) and disease-free survival (DFS), we employed the multivariable COX regression and LASSO regression analysis. Additionally, Spearman correlation analysis, logistic regression, and receiver-operating characteristic (ROC) curve analysis were conducted to assess the predictive value of the CK-MB/CK ratio for postoperative liver metastasis.

**Results:**

Cox regression analysis revealed that the CK-MB/CK ratio was a stable risk factors for OS (HR = 3.82, *p* < 0.001) and DFS (HR = 2.31, *p* < 0.001). To distinguish hepatic metastases after surgery, the ROC area under the curve of CK-MB/CK was 0.697 (*p* < 0.001), and the optimal cut-off value determined by the Youden index was 0.347.

**Conclusions:**

Postoperative abnormal CK-MB/CK ratio predicts worse prognosis in CRC patients after radical resection and serves as a useful biomarker for detecting postoperative liver metastasis.

**Supplementary Information:**

The online version contains supplementary material available at 10.1186/s12885-024-12307-5.

## Background

Colorectal cancer (CRC) is one of the most lethal malignancies worldwide. According to global cancer estimates, CRC ranks third in incidence but second in mortality [1]. In China, both the incidence and mortality have increased over the past decade [1], and the 1-year, 3-year, and 5-year survival rates were 79%, 72%, and 62% respectively [2]. Currently, radical resection is a primary treatment for this disease. However, post-surgery, 30% of stage II and 50–60% of stage III patients develop recurrence or metastases within 5 years [3, 4]. Up to 50% of patients with initially localized disease will develop metastases [5]. For this reason, recurrence and metastases post-surgery remain the leading causes of treatment failure and cancer-related death [4, 6]. It is urgent to explore effective postoperative surveillance biomarkers to reduce the huge economic burden on society of this disease.

**Fig. 1:**
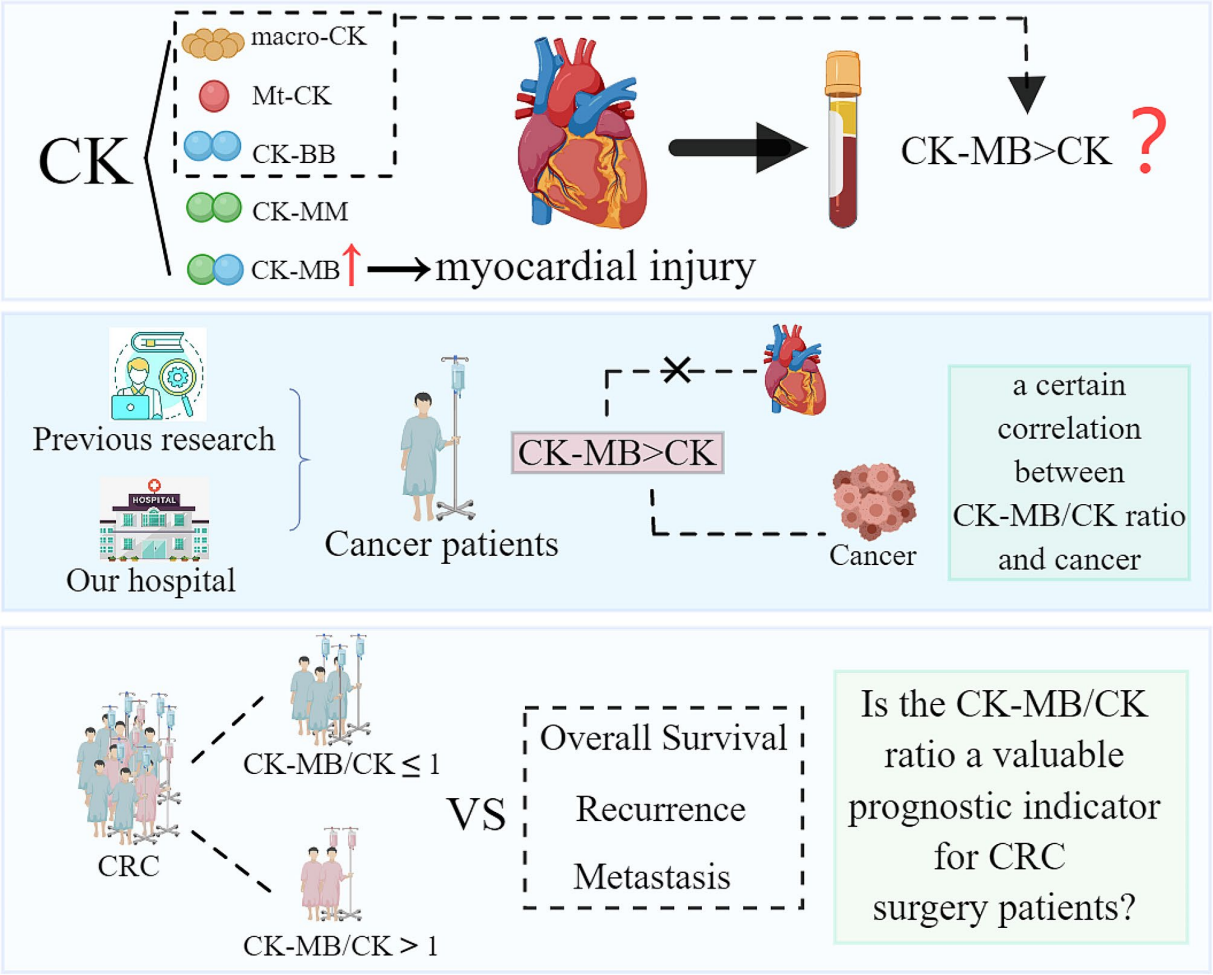
Research background and main research questions. CK, Creatine Kinase; Macro-CK, Macro creatine kinase; Mt-CK, Mitochondrial creatine kinase; CK-BB, Creatine kinase type B; CK-MM, Creatine kinase type M; CK-MB, Creatine kinase type M and B; CRC, Colorectal cancer

Creatine kinase (CK) serves as a central controller of cellular energy homeostasis and is widely distributed in body tissues. It reversibly catalyzes the formation of creatine phosphate and adenosine diphosphate from creatine and adenosine triphosphate, playing an important role in tissues with high energy demands [7]. CK exists in three major isoenzymes: creatine kinase type M (CK-MM), creatine kinase type B (CK-BB), and creatine kinase type M and B (CK-MB) [8]. Additionally, mitochondrial creatine kinase (Mt-CK) and macro creatine kinase (macro-CK) were also reported as different forms of CK in vivo [9, 10]. CK-MB is primarily employed for diagnosing myocardial injury [11, 12]. When myocardial cells are damaged, serum CK-MB activity levels increase. For the determination of CK-MB, the immunoinhibition method is utilized most commonly. However, the detection of CK-MB activity by immunosuppressive assays might be influenced by the presence of CK isoenzymes in the serum [13]. In cases where CK-BB [14] or macro-CK [7, 15] is present in the serum of tumor patients, even without myocardial injury, abnormal results indicating increased CK-MB activity may occur [14, 16]. In situations where a significant amount of CK-BB and macro-CK is present, abnormal results may occur where CK-MB exceeds the total CK ratio (CK-MB > CK) [17]. Previous studies have highlighted CRC as the most common malignancy with abnormal CK-MB > CK results [13, 18] (Fig. 1).

We frequently observed abnormal testing results of CK-MB > CK (defined as CK-MB/CK > 1) in CRC patients who did not show evidence of myocardial injury. This phenomenon raises speculation that other potential pathological factors may lead to the elevated expression of CK-BB and macro-CK in the serum of tumor patients. However, the exact role of these factors in tumors remains not fully understood. Earlier studies demonstrated the prognostic significance of macro-CK detected in the serum of patients with hepatocellular carcinoma [7, 19] and breast cancer [20] had prognostic significance. These findings suggest that CK-BB or macro-CK activity might serve as a potential biomarker in cancer patients [21, 22]. The CK-MB/CK is an easily available indicator and could be clinically utilized as a primary screening tool for cancer [13]. Given the emerging link between increased CK-MB activity detected by the immunosuppressive method in cancer patients and their poor prognosis [8], serum CK-MB/CK might ultimately serve as a prognostic marker, an issue that deserves further investigation. Presently, no studies have reported the role of abnormally elevated CK-MB activity in the prognosis of patients with CRC. Therefore, this study is the first to explore the role of CK-MB/CK in postoperative patients with CRC (Fig. 1).

In this retrospective study, we analyzed 1272 stage I-III CRC patients with the aim of identifying clinical factors associated with abnormal CK-MB > CK results. Additionally, we sought to evaluate the prognostic impact of CK-MB/CK on survival, recurrence, and metastasis.

## Patients and methods

### Study population and design

The study population comprised CRC patients who were hospitalized in the Sichuan cancer hospital between January 2017 and May 2021. The inclusion and exclusion criteria were as follows:

Inclusion criteria: (1) patients with CRC diagnosed by histopathology; (2) patients with stages I-III as determined by the tumor-node-metastasis (TNM) stages established by the American Joint Committee on Cancer (AJCC); (3) patients with completed resection of the primary tumor; (4) patients with postoperative follow-up data; (5) patients with available postoperative serum CK-MB and total CK measurements.

Exclusion criteria: (1) patients with a history of other malignancies; (2) patients with an unknown pathological stage; (3) patients classified as Tis by histopathology; (4) patients with stage IV; (5) patients with myocardial injury caused by different reasons, including cardiovascular complications [23], COVID-19 infection, myocardial injury after noncardiac surgery [24], etc. Based on clinical manifestations and laboratory evidence, such as chest pain, electrocardiogram examination, cardiac troponin, myoglobin, and brain natriuretic peptide, etc. [25]; (6) patients with uncompleted data.

Finally, a total of 1272 patients were enrolled in the retrospective study. We extracted necessary data from medical records, such as demographics, clinical data, laboratory results, histological findings, follow-up, elapsed time to either local or distant recurrence, the short and long term outcome as well as survival, etc. Follow-up data included prognosis, mortality status, and recurrence after the surgery.

According to whether the CK-MB and CK test results were abnormal, patients were divided into two groups: the abnormal group (CK-MB > CK, i.e., CK-MB/CK > 1) and the normal group (CK-MB ≤ CK, i.e., CK-MB/CK ≤ 1).

### Serum analyses

The CK-MB/CK ratio in our study was measured within 3 days after surgery. CK-MB and CK were detected from the same blood sample simultaneously, using the same detection instrument. In the case of several measurements showing CK-MB > CK, the first value was considered. We took the first test result because through it we were able to identify patients’ health status earlier after surgery.

Serum total CK and CK-MB concentrations were measured by Mindray BS-2000 M automatic biochemical analyzer (Mindray Bio-Medical Electronics Corporation, Shenzhen, Guangdong, China), using the phosphocreatine substrate method and the immunosuppression method, respectively. The reference range of CK-MB was set at ≤ 25 U/L. The reference range of total CK was 24 ∼ 194 U/L for males and 24 ∼ 170U/L for females. The serum carbohydrate antigen 50 (CA50) and carbohydrate antigen 242 (CA242) concentrations were measured by the Mindray CL-6000i chemiluminescence analyzer (Mindray Bio‐Medical Electronics Corporation, Shenzhen, Guangdong, China). The serum carbohydrate antigen 724 (CA72-4), carbohydrate antigen 199 (CA19-9), and Carcinoembryonic antigen (CEA) concentrations were detected using the Roche e411 electrochemiluminescence analyzer (Roche Diagnostics, Mannheim, Germany).

### Statistical analysis

The sample size was estimated using PASS software. The downSample function was employed to address unbalanced data. The distribution of the data was assessed using histograms, boxplots, normal probability plots, and the Shapiro-Wilk test, which is suitable for small samples. Normally distributed data were presented as the mean and standard deviation, while non-normally distributed data were represented using the median and interquartile range. Categorical variables were expressed as frequencies and percentages. When comparing clinical data differences between the normal and abnormal groups, Pearson’s chi-square test or Fisher’s exact test was utilized for categorical variables, and the t-test was employed for numerical variables [26].

Overall Survival (OS) was calculated from the date of the surgical operation until death or the deadline for follow-up. Disease-free Survival (DFS) was calculated from the date of the surgical operation until the date of tumor recurrence, metastasis, the appearance of tumor-related diseases, or the deadline for follow-up. The censoring was defined as follows: patients who did not experience death, recurrence, or metastasis during the period from the surgery date to the follow-up deadline of this study, but had non-tumor-related factors leading to death or loss to follow-up. Censored individuals have their last observed times recorded and are identified as censored observations. We assumed that censoring was independent of survival events. We utilized the Cox proportional hazards model and Kaplan-Meier survival curves to effectively handle and incorporate censored observations, ensuring that they do not impact the accuracy of survival analysis. The Kaplan-Meier method was employed to estimate OS and DFS. The median follow-up time was calculated using the Reverse Kaplan-Meier method, which considers the weight of the study subjects with the outcome event, yielding more accurate results. Differences in survival were assessed with the log-rank test. The LASSO regression model and multivariable COX proportional hazards model were utilized to analyze the influencing factors related to OS and DFS. Covariates, including age, gender, primary site, differentiation, TNM stage (AJCC 8th), neoadjuvant therapy, CEA, CA50, CA19-9, CA242, and CA72-4, were adjusted to evaluate the impact of CK-MB/CK on survival. We employed the Schoenfeld residual test to assess the proportional hazards assumption of the Cox model and visually examined it using residual plots. Martingale residual scatter plots were used to evaluate the linearity assumption of continuous variables for Cox regression.

Spearman’s correlation test was used to analyze the correlation between biomarkers and postoperative metastasis sites. When comparing the differences in clinical data between patients with hepatic metastases and patients without hepatic metastases, Pearson’s chi-square test was used for categorical variables and t-test was used for numerical variables. When analyzing the impact of CK-MB/CK on liver metastasis, a multivariable logistic regression model was used, with adjustment variables including age, gender, primary site, differentiation, TNM stage (AJCC 8th), and neoadjuvant therapy. Besides, the Bootstrap method and the Hosmer-Lemeshow goodness-of-fit test were performed to evaluate the performance metrics of the logistic regression model. Receiver operating characteristic (ROC) curves were used to evaluate the ability of CK-MB/CK to distinguish patients with liver metastasis and without liver metastasis after surgery. We calculated the area under the ROC curve (AUC) and using the Bootstrap method to evaluate the corrected AUC. The optimal cut-off value was determined by the Youden index (J) from the ROC curve [27]. Youden index (J) is defined as the maximum vertical distance between the ROC curve and the diagonal or chance line and is calculated as J = maximum (sensitivity + specificity − 1) [28].

A two-sided *P*-value of less than 0.05 was considered statistically significant, and 95% confidence interval (CI) was calculated. All statistical analyses were performed with the IBM SPSS Statistics Program (version 24), R version 4.3.1 (R Foundation for Statistical Computing, Vienna, Austria. http://www.r-project.org accessed on June 16, 2023), MedCalc 12.7 (MedCalc Software, Ostend, Belgium), and GraphPad Prism software (version 9.5).

## Results

### Subjects

**Fig. 2:**
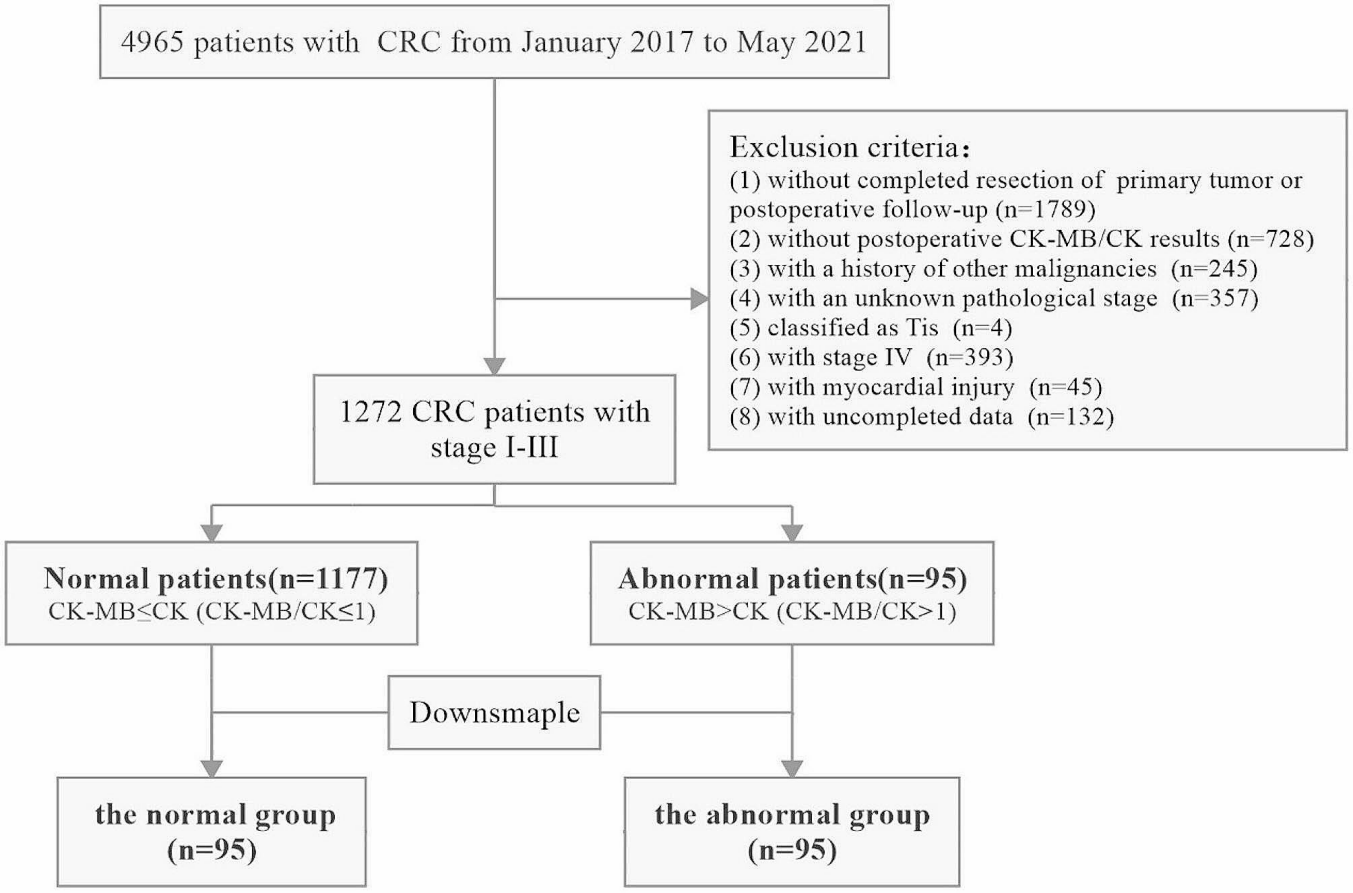
Enrollment flow chart of eligible patients in the present study. CRC, colorectal cancer; CK-MB, creatine kinase type M and B; CK, creatine kinase

From January 2017 to May 2021, 4965 CRC patients were treated in our hospital. 3693 patients who didn’t meet the inclusion and exclusion criteria were excluded, including 1789 patients without completed resection of the primary tumor or postoperative follow-up, 728 patients without postoperative serum CK-MB/CK results, 245 patients with a history of other malignancies, 357 patients with an unknown pathological stage, 4 patients classified as Tis, 393 patients with stage IV, 45 patients with myocardial injury, and 132 patients with uncompleted data. Finally, 1272 patients were included in our study. Among them, only 95 patients had abnormal results. Since there was an obvious imbalance in the sample size between the normal patients and the abnormal patients, we used the downsample method to process unbalanced samples. Finally, 95 cases in the normal group and 95 cases in the abnormal group were included in our analysis. The study size and flowchart were described in Fig. 2. Since the first patient was included, we counted the number and proportion of patients included each year. The results showed that 72% of patients were included in 2017–2019 (Supplementary Table 1). The median follow-up time of our study population was 32.6 months (95% CI, 30.96–34.24) (Supplementary Table 2).

### Clinicopathologic characteristics of patients in the normal group and abnormal group

**Fig. 3:**
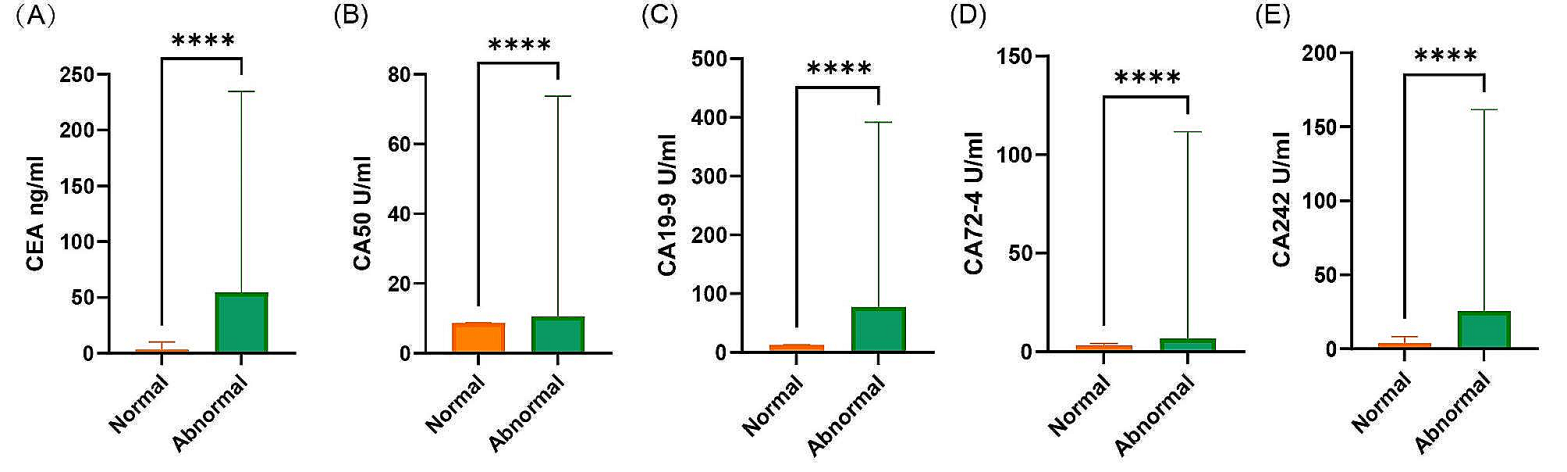
Comparison of tumor biomarkers between the normal group and abnormal group. *P* values from the t-test. Abbreviations: CEA, carcinoembryonic antigen; CA, carbohydrate antigen

Table 1 summarizes the clinical and pathological variables of CRC patients in the abnormal and normal groups. There were no significant differences in age, gender, primary site of cancer, depth of tumor, or neoadjuvant therapy. However, other variables indicating disease severity, such as tumor differentiation, TNM stage (AJCC 8th), and regional lymph nodes were significantly different between the two groups. We analyzed the expression of clinical commonly used tumor markers in patients between the two groups. The results showed that CEA, CA50, CA19-9, CA242, and CA72-4 had significant differences. The patients in the abnormal group had higher levels of serum CEA, CA50, CA19-9, CA242, and CA72-4 (all *P* < 0.001) (Fig. 3). Besides, we compared the correlation between CK-MB/CK and these tumor biomarkers. The results showed that serum expression levels of CK-MB/CK were significantly positively correlated with these biomarkers, whether in the total study population or subgroups of different stages, gender, and age ranges(≤ 60 or >60 years old) (Supplementary Table 3).

**Table 1:** Clinicopathologic characteristics of patients with normal and abnormal postoperative serum CK-MB/CK concentrations

Parameter	Normal group	Abnormal group	*P* value
	*n* = 95	*n* = 95	
Age [years, mean(sd)]	60.95 (11.5)	58.19 (13.1)	0.12
Gender [n(%)]			0.14
Male	64 (67)	53 (56)	
Female	31 (33)	42 (44)	
Primary site [n(%)]			0.11
Colon	37 (39)	49 (52)	
Rectum	58 (61)	46 (48)	
Differentiation [n(%)]			< 0.001
Poor	7 (7.3)	22 (23)	
Moderate	78 (82)	52 (55)	
Well	10 (11)	21 (22)	
TNM stage (AJCC 8th) [n(%)]			0.014
I	16 (17)	16 (17)	
II	41 (43)	23 (24)	
III	38 (40)	56 (59)	
Depth of tumor [n(%)]			0.6
T1/2	20 (21)	16 (17)	
T3/4	75 (79)	79 (83)	
Regional lymph nodes [n(%)]			0.013
N0	57 (60)	39 (41)	
N+	38 (40)	56 (59)	
Neoadjuvant therapy [n(%)]			0.4
No	72 (76)	66 (69)	
Yes	23 (24)	29 (31)	

The *P* values of age was performed using the t-test. The *P* values of gender, primary site, differentiation, TNM stage, depth of tumor, regional lymph nodes, and neoadjuvant therapy were performed using the chi-square test. Abbreviations: AJCC, American Joint Committee on Cancer; CK-MB, creatine kinase type M and B; CK, creatine kinase

### Comparing the prognosis of OS and DFS between the abnormal group and the normal group

**Fig. 4:**
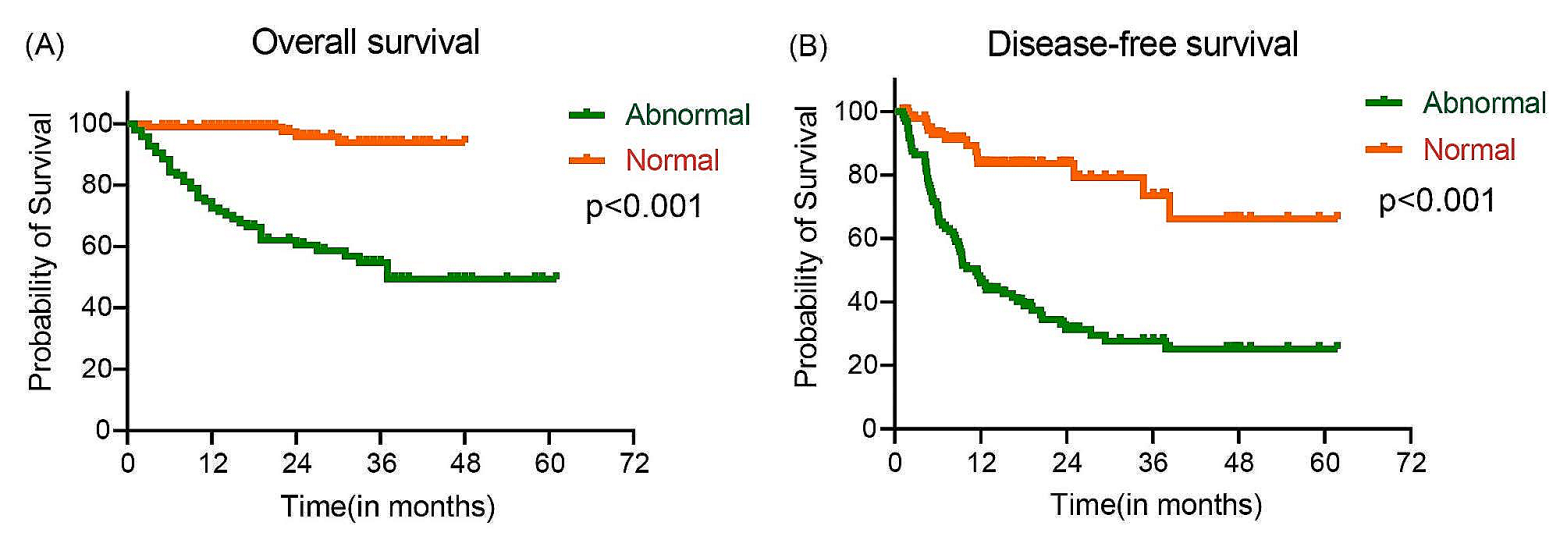
Kaplan-Meier plots of the abnormal group and the normal group on overall survival (**A**) and disease-free survival (**B**). *P* values from log-rank test. Abbreviations: CK-MB, creatine kinase type M and B; CK, creatine kinase

**Fig. 5:**
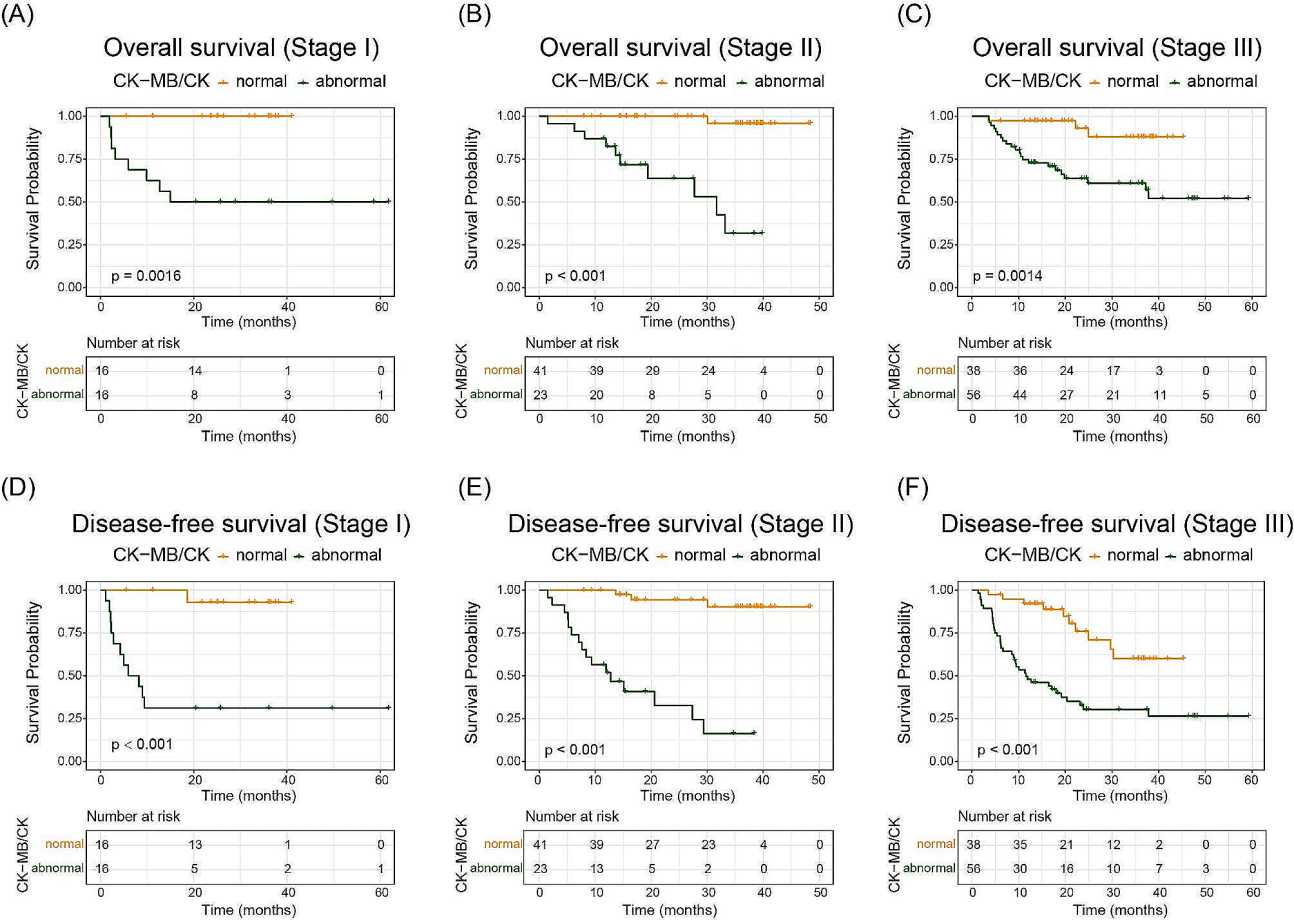
Kaplan-Meier plots of abnormal and normal CRC patients in different stage on overall survival (**A-C**) and disease-free survival (**D-F**). *P* values from log-rank test. Abbreviations: CK-MB, creatine kinase type M and B; CK, creatine kinase

Patients with abnormal postoperative CK-MB/CK showed impaired OS compared with patients with normal CK-MB/CK (38.05 months vs. 46.76 months; HR, 12.48, 95%CI, 6.88–22.61; *P* < 0.001). On the other hand, patients with abnormal CK-MB/CK had significantly shorter DFS (23.38 months vs. 42.68 months; HR, 4.64, 95%CI, 2.99– 7.22; *P* < 0.001) than patients with normal CK-MB/CK (Fig. 4). In different TNM stages, the abnormal group also showed worse OS and DFS than the normal group (Fig. 5).

### LASSO and multivariable COX proportional hazards regression analysis of prognostic factors for overall survival and disease-free survival

Initially, we utilized LASSO and multivariable COX proportional hazards regression models to investigate the association of CK-MB/CK with patient prognosis, thereby clarifying the potential clinical value of CK-MB/CK. The results from multivariable COX regression analysis revealed that, even after adjusting for confounding factors such as age, gender, primary site, differentiation, TNM stage (AJCC 8th), neoadjuvant therapy, CEA, CA50, CA19-9, CA242, and CA72-4, CK-MB/CK continued to exhibit a significant relationship with OS (HR, 3.82; 95% CI, 1.98–7.40; *p* < 0.001) and DFS (HR, 2.31; 95% CI, 1.50–3.57; *p* < 0.001) (Table 2). By employing the Schoenfeld residual test to assess the proportional hazards assumption of the Cox regression, the results indicated no significant relationship between residuals and time in our analysis, supporting the proportional hazards assumption (Supplementary Table 4). Additionally, the residual plot demonstrated that the variation of residuals over time followed a random pattern with no discernible systematic trend (Supplementary Fig. 1). We utilized Martingale residual scatter plots to evaluate the linearity assumption, which results indicated that continuous variables fit the linear relationship required for Cox regression. The results for OS and DFS were shown in Supplementary Fig. 2 and Supplementary Fig. 3, respectively.

**Table 2:** Multivariable Cox regression analyses of prognostic factors for overall survival and disease-free survival

	Overall survival		Disease-free survival	
	HR(95%CI)	*P*	HR(95%CI)	*P*
Age	1.01(0.98–1.04)	0.4	0.99(0.968–1.012)	0.4
Gender (Female)	0.85(0.43–1.69)	0.6	1.06(0.64–1.75)	0.8
Primary site (Rectum)	1.55(0.58–4.13)	0.4	1.84(0.87–3.90)	0.1
Differentiation				
Medium	1.47(0.55–3.90)	0.4	2.48(1.16–5.30)	0.02
High	0.84(0.38–1.82)	0.7	0.85(0.45–1.59)	0.6
TNM stage (AJCC 8th)				
II	2.19(0.83–5.75)	0.1	2.48(1.10–5.62)	0.03
III	1.19(0.46–3.11)	0.7	1.30(0.64–2.68)	0.5
Neoadjuvant therapy	0.37(0.12–1.18)	0.09	0.25(0.10–0.64)	0.004
CEA^*^	1.000(0.999–1.002)	0.4	1.001(1.000-1.002)	0.01
CA50^*^	0.997(0.992–1.002)	0.2	0.998(0.994–1.001)	0.2
CA19-9^*^	1.000(0.998–1.001)	0.6	1.000(0.999–1.001)	0.8
CA242^*^	1.003(0.997–1.009)	0.3	1.001(0.996–1.006)	0.7
CA72-4^*^	1.005(1.001–1.008)	0.01	1.005(1.002–1.007)	0.002
CK-MB/CK^*^	3.82(1.98–7.40)	< 0.001	2.31(1.50–3.57)	< 0.001

^*^: These variables were inputted as continuous numeric variables. Adjusted factors: age, gender, primary site, differentiation, TNM stage (AJCC 8th), neoadjuvant therapy, CEA, CA50, CA19-9, CA242, and CA72-4. Abbreviations: HR, hazard ratio; CI, confidence interval; CEA, carcinoembryonic antigen; CA, carbohydrate antigen; CK-MB, creatine kinase type M and B; CK, creatine kinase

**Fig. 6:**
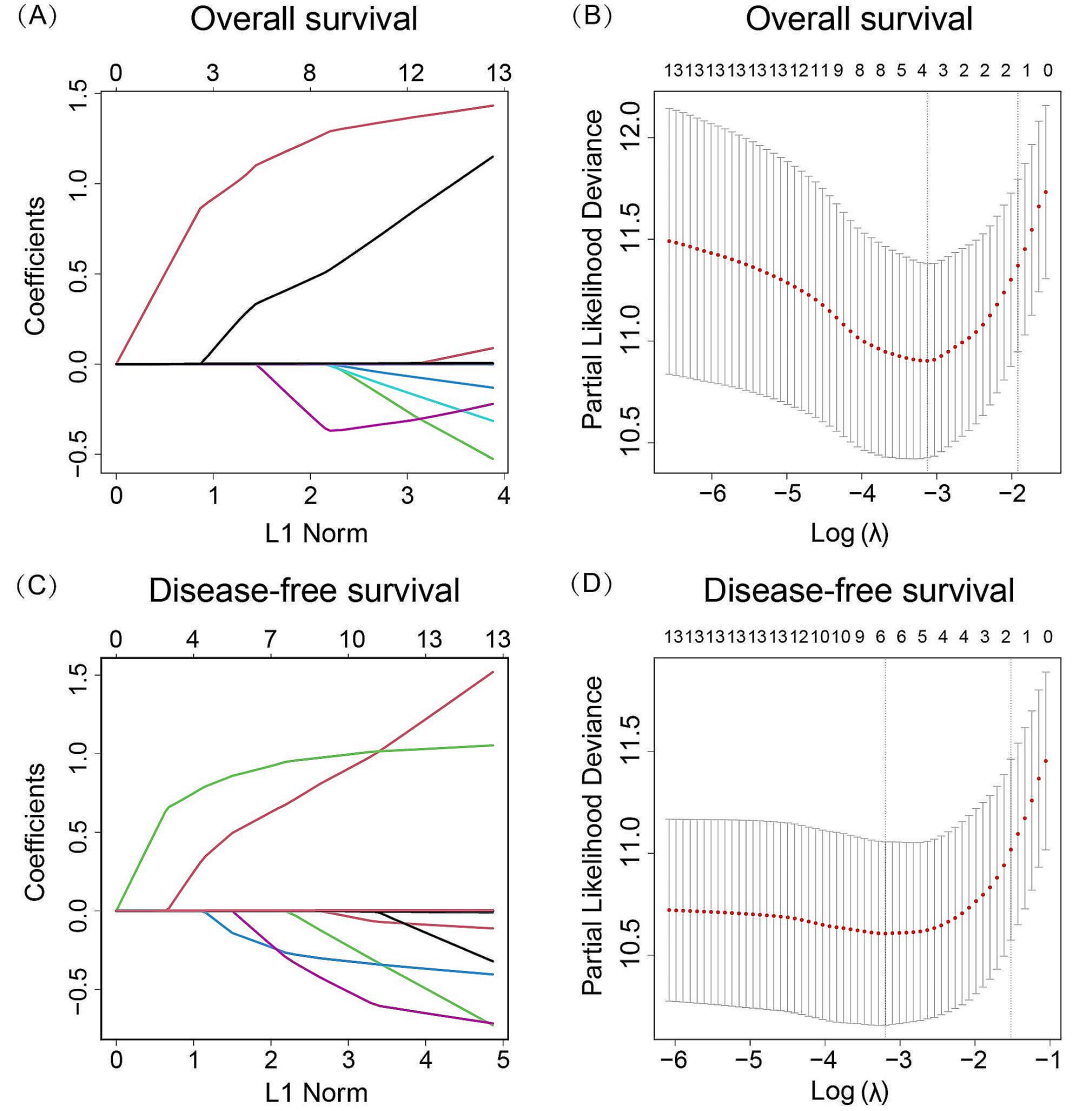
Screening influencing factors for overall survival and disease-free survival with the least absolute shrinkage through the Lasso regression model. (**A-B**) Lasso regression and Cross-validation of overall survival. (**C-D**) Lasso regression and Cross-validation of disease-free survival. Adjusted factors: age, gender, primary site, differentiation, TNM stage (AJCC 8th), neoadjuvant therapy, CEA, CA50, CA19-9, CA242, and CA72-4

In the LASSO regression model, the results indicated that CK-MB/CK, CA72-4, and neoadjuvant therapy were significantly related to OS. Additionally, CK-MB/CK, CA72-4, neoadjuvant therapy, CEA, and differentiation showed significant associations with DFS (Fig. 6). All the above results collectively demonstrated that CK-MB/CK was a stable influencing factor for OS and DFS in patients undergoing CRC surgery.

Through the above analysis, we found that the abnormal group had a worse prognosis than the normal group. Our interest lies in exploring the specific factors that impact prognosis. Consequently, we sought to identify disease progression factors associated with abnormal CK-MB/CK ratio. However, is CK-MB/CK directly related to postoperative disease recurrence and metastasis? Therefore, we analyzed the 1272 CRC patients collected. In the abnormal group, a total of 62 (65%) patients developed a recurrence of the disease after surgery. One patient developed local recurrence (1.1%), while the remaining 61 developed distant metastases namely: liver (*n* = 32, 33.7%), lung (*n* = 9, 9.5%), bone (*n* = 6, 6.3%), and other sites (*n* = 18, 18.9%). In the normal group, a total of 114(9.7%) patients had a recurrence or distant metastasis, including local recurrence (*n* = 10, 0.8%), liver (*n* = 30, 3.3%), lung (*n* = 49, 4.2%), bone (*n* = 13, 1.1%), and other sites (*n* = 19, 1.6%) (Table 3). Besides, by correlation analysis, we found that CA19-9, CA242, and CK-MB/CK were correlated significantly with liver metastasis (Spearman’s Rho 0.208, *P* < 0.001; Rho 0.149, *P* < 0.001; Rho 0.175, *P* < 0.001, respectively), but weakly correlated with lung metastasis (Table 4).

**Table 3:** The locoregional or systemic recurrence after operation in the abnormal group and the normal group

	Normal group	Abnormal group	*P* value
Locoregional recurrence [n(%)]	10(0.8)	1(1.1)	0.6
Systemic recurrence			
Hepatic [n(%)]	39(3.3)	32(33.7)	< 0.001
Pulmonary [n(%)]	49(4.2)	9(9.5)	0.03
Bone [n(%)]	13(1.1)	6(6.3)	0.002
Other^*^ [n(%)]	19(1.6)	18(18.9)	< 0.001

^*^: Other anatomic sites included the abdominal cavity, pelvic cavity, adnexa, ovary, uterus, and bladder. *P* values from the Pearson’s chi-square test, or Fisher’s exact test, where appropriate

**Table 4:** The correlation analysis of biomarkers and systemic recurrence after surgery. Rho and *P* values from Spearman’s correlation test

Biomarkers		Hepatic	Pulmonary	Bone	Other^*^
CA19-9	Rho	0.208	0.022	0.030	0.049
	*P*	< 0.001	0.5	0.4	0.1
CA242	Rho	0.149	0.017	0.017	0.033
	*P*	< 0.001	0.5	0.6	0.2
CK-MB/CK	Rho	0.175	0.041	0.061	0.138
	*P*	< 0.001	0.1	0.03	< 0.001

^*^: Other anatomic sites included the abdominal cavity, pelvic cavity, adnexa, ovary, uterus, and bladder. CK-MB, creatine kinase type M and B; CK, creatine kinase

### Biomarkers in hepatic metastases versus non-hepatic metastases patients after surgery

Thus, we directed our focus towards liver metastasis. Among the 869 cancer patients who had the results of CA19-9, CA242, and CK-MB/CK, we compared the serum expression levels of those biomarkers between the hepatic metastases and non-hepatic metastases patients after surgery. The results showed that CA19-9, CA242, and CK-MB/CK all had significant differences between the two groups. Compared with the non-hepatic metastases patients, the hepatic metastases had higher CA19-9 (median 35.46 U/ml and 12.22 U/ml, respectively; *P* < 0.001), CA242 (median 12.27 U/ml and 5.81 U/ml, respectively; *P* < 0.001), and CK-MB/CK (median 0.35 and 0.09, respectively; *P* < 0.001) (Table 5).

**Table 5:** The serum expression levels of biomarkers in the hepatic metastases and non-hepatic metastases patients after surgery

Parameter	Hepatic metastases (*n* = 68)	Non-hepatic metastases (*n* = 801)	*P* value
Age [years, median(Q1, Q3)]	57 (51.75, 66.25)	61 (52, 70)	0.2
Gender [n(%)]			0.03
Male	47 (69)	440 (55)	
Female	21 (31)	361 (45)	
Primary site [n(%)]			0.02
Colon	21 (31)	367 (46)	
Rectum	47 (69)	434 (54)	
Differentiation [n(%)]			0.1
Poor	10 (15)	118 (15)	
Moderate	47 (69)	611 (76)	
Well	11 (16)	72 (9.0)	
TNM stage [n(%)]			0.1
I	8 (12)	108 (13)	
II	15 (22)	268 (33)	
III	45 (66)	425 (54)	
Depth of tumor [n(%)]			0.3
T1/2	10 (15)	137 (17)	
T3/4	58 (85)	664 (83)	
Regional lymph nodes [n(%)]			0.1
N0	23 (34)	376 (47)	
N+	45 (66)	425 (53)	
Neoadjuvant therapy [n(%)]			< 0.001
No	34 (50)	587 (73)	
Yes	34 (50)	214 (27)	
CA19-9(U/ml)	35.46(14.69–226.50)	12.22(6.67–23.91)	< 0.001
CA242(U/ml)	12.27(4.40-85.91)	5.81(2.71–11.94)	< 0.001
CK-MB/CK	0.35(0.07–1.22)	0.09(0.05–0.18)	< 0.001

The *P* values of age, CA19-9, CA242, and CK-MB/CK were performed using the t-test. The *P* values of gender, primary site, differentiation, TNM stage, depth of tumor, regional lymph nodes, and neoadjuvant therapy were performed using the chi-square test. CA, carbohydrate antigen; CK-MB, creatine kinase type M and B; CK, creatine kinase

### Performance of CK-MB/CK to predict hepatic metastasis

To evaluate the potential predictive value of the serum CK-MB/CK ratio for liver metastasis following CRC surgery, we initially conducted a multivariable logistic regression analysis, which was crucial for our study. Because it might explain the association of the prognosis, from the perspective of liver metastasis progression. After adjusting for variables including age, gender, primary site, differentiation, TNM stage (AJCC 8th), and neoadjuvant therapy, CK-MB/CK and CA19-9 still exhibited significant predictive ability for postoperative liver metastasis, with respective odds ratio values of 5.46 and 1.003 (Table 6).

**Table 6:** The multivariable logistic regression model for predicting hepatic metastasis in CRC patients after surgery

Biomarkers	*P* value	Odds ratio	95%CI
CK-MB/CK	< 0.001	5.46	2.83–10.54
CA19-9	0.001	1.003	1.002–1.005
CA242	0.1	0.99	0.98-1.00

Model is controlled for age, gender, primary site, differentiation, TNM stage (AJCC 8th), and neoadjuvant therapy. CA, carbohydrate antigen; CK-MB, creatine kinase type M and B; CK, creatine kinase

Subsequently, all observational factors, including age, gender, TNM stage, CA19-9, CA242, and CK-MB/CK, etc. were incorporated into the analysis to construct the logistic regression model and calculate the AUC, resulting in 0.849 [95%CI, 0.800-0.898]. Internal validation using the Bootstrap method showed that the corrected AUC of the model was 0.791.

To ensure the reliability of our research findings, we comprehensively evaluated the performance metrics of the logistic model. We utilized the Hosmer-Lemeshow goodness-of-fit test to evaluate the calibration of the logistic regression model. The results indicated χ²=6.94, *P* = 0.54, suggesting that the difference between the predicted values and the actual values of our logistic regression model was not statistically significant, indicating good consistency. Additionally, we assessed the linear trend by plotting scatterplots, which demonstrated that all continuous variables met the linearity assumption conditions (Supplementary Fig. 4). Thus, we preliminarily established the stable impact of CK-MB/CK on liver metastasis.

**Fig. 7:**
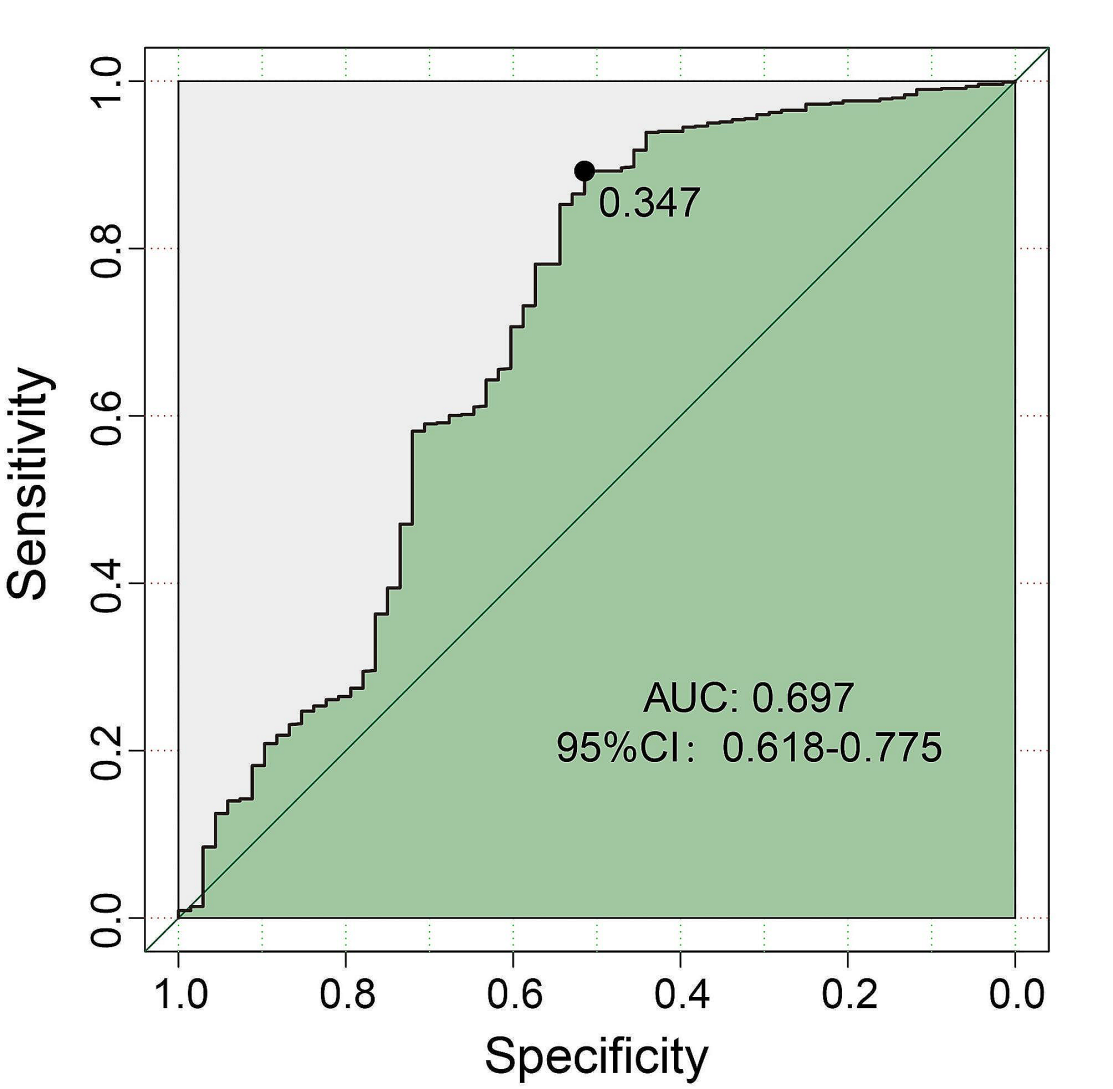
The ROC curve of the CK-MB/CK ratio to distinguish hepatic metastases and non-hepatic metastases after surgery. AUC, area under the curve; CI, confidence interval; CK-MB, creatine kinase type M and B; CK, creatine kinase; ROC, receiver-operating characteristic

Then, using CK-MB/CK as a single indicator for liver metastasis identification, we plotted the ROC curve. When distinguishing patients with hepatic metastases from those without, the AUC for serum CK-MB/CK was 0.697 [95%CI, 0.618–0.775; *P* < 0.001]. Internal validation using the Bootstrap method showed that the corrected AUC of CK-MB/CK was 0.620. The optimal cut-off value determined by the Youden index was 0.347 (Fig. 7).

## Discussion

The global incidence rate of cancer has been on the rise [29]. Consequently, there is a pressing need to establish effective biomarkers for monitoring cancer risk. However, novel tumor markers are often initially cost-ineffective, leading to limited adoption in clinical laboratories. Therefore, easily accessible biomarkers aiding in the evaluation and diagnosis of cancer would be valuable and practical in clinical practice.

The CK-MB/CK ratio, derived from commonly used data on CK-MB activity and total CK activity for myocardial injury assessment [11], serves as a widely available indicator applicable in clinical laboratories for cancer assessment. Numerous studies have demonstrated a specific association between a higher CK-MB/CK ratio and certain malignancies [18], such as CRC, lung cancer, pancreatic cancer [30], and hepatocellular carcinoma [13]. However, the specific relationship between a tumor and its pathogenesis remains unclear. Within our research institution, we noted a significant number of CRC patients with abnormal CK-MB > CK testing results, consistent with findings reported by Chang C C et al. [18]. Consequently, our study aims to investigate the relationship between abnormal CK-MB > CK results and CRC patients.

By comparing the clinical data of CRC patients with abnormal CK-MB/CK and those with normal CK-MB/CK after radical resection, we aimed to identify clinical features or laboratory-related test indicators associated with this difference. The results revealed significant differences in prognosis between the normal and abnormal groups. And these differences were still significant in different TNM stages. The Kaplan-Meier results showed that patients with abnormal CK-MB/CK had worse OS and DFS. Existing research indicates that regional lymph nodes play a crucial role as a prognostic factor for survival after surgery [31]. However, in our study, differences in survival were not attributed to regional lymph nodes. We hypothesized that CK-MB/CK might serve as a potential new prognostic indicator for CRC patients following radical surgery.

Furthermore, we observed significantly higher serum levels of tumor biomarkers (CEA, CA50, CA19-9, CA242, and CA72-4) during postoperative follow-up in the abnormal group. CA72-4 has been recognized as a valuable indicator for predicting tumor recurrence survival in non-metastatic CRC patients undergoing surgery [32]. In radically operated CRC patients, postoperatively elevated CEA or CA19-9 levels may indicate a high risk of relapse [33–36]. Thus, we speculate that CK-MB/CK might exhibit synergistic effects with these tumor markers. To mitigate the influence of confounding factors, we conducted further analyses using multivariable COX regression and LASSO regression. The results demonstrated that even after adjusting for the influence of covariates, CK-MB/CK remains a stable factor affecting prognosis. The HR results of CK-MB/CK for COX regression analysis were 3.82 for OS and 2.31 for DFS. The results were acceptable and consistent with clinical reality. Because, in clinical practice, it was extremely difficult for the CK-MB/CK ratio to increase by a single unit. Typically, the ratio fluctuates at the level of 0.01 in abnormal patients, as determined by the detection principles of CK-MB and CK. After surgery, patients with abnormal CK-MB/CK results had a shorter survival time, and higher risk of recurrence and distant metastasis than those with the normal results.

To delve into the specific role of CK-MB/CK in postoperative follow-up, we conducted a detailed analysis of the relationship between CK-MB/CK and metastasis. By examining the recurrence and metastasis scenarios in both the abnormal and normal groups, we identified no significant differences in locoregional recurrence. However, noteworthy differences emerged in distant metastasis, particularly in liver, lung, and bone metastases. It is conceivable that postoperative CK-MB/CK may be associated with specific organs or tissues prone to distant metastasis. Previous studies have suggested the possibility of distinguishing tissue damage by measuring the activity of different CK subtypes [13]. Kovar FM et al. [13] also noted that the CK-MB/CK ratio might reflect the extent of inner organ damage, often representing a major prognostic risk factor. Consequently, we further explored the relationship between CK-MB/CK and metastasis sites. Correlation analysis revealed significant associations between CA19-9, CA242, and CK-MB/CK with liver metastasis. However, correlations with lung and bone metastases were found to be weak.

To confirm the relationship between postoperative CK-MB/CK and liver metastasis, the 869 patients with CK-MB/CK, CA19-9, and CA242 detection results were analyzed separately. The serum expression levels of these biomarkers were compared between patients with liver metastasis and patients without liver metastasis after the operation. We found that patients with liver metastasis had higher levels of serum CK-MB/CK, CA19-9, and CA242. This was consistent with the previous research findings of Kovar FM et al. [13] and Chang C C et al. [18], that the CK-MB/CK ratio was markedly higher in liver metastasis than in non-liver metastasis. Furthermore, it was recently reported that higher CK-BB activity was associated with human liver metastasis [37], and serum Mt-CK activity increases in patients with liver cancer [7], which was partially compatible with and illustrates our results. However the underlined mechanism remained unknown and could be worth further research. Could this significant difference in expression level be used as an effective criterion to distinguish between postoperative liver metastasis and non-liver metastasis? Therefore, we further analyzed the ROC curve [38] and the AUC of CK-MB/CK was 0.697.

There were several limitations in our study. All subjects were stage I-III patients without distant metastasis. In stage IV patients with distant metastasis, the prognostic value of CK-MB/CK needs further exploration. Additionally, our study didn’t rule out clinical conditions that could lead to high CK-MB/CK ratio rather than neoplasms, including brain injury, skeletal muscle injury, polytraumatism [13], or severe shock syndrome because cancer patients usually had other complications. These comorbidities might also cause abnormal CK-MB/CK ratio. In addition, the abnormal CK-MB activity estimated by the immunosuppression method has not been further determined. It might be caused by an increase in specific CK isoenzyme subtypes, which would have a specific role in CRC and need to be further clarified.

In conclusion, our results demonstrate that postoperative CK-MB/CK ratio proves beneficial in evaluating prognosis of OS and DFS for CRC patients after surgery. Additionally, the serum CK-MB/CK ratio serves as an useful biomarker for distinguishing postoperative liver metastasis.

### Electronic supplementary material

Below is the link to the electronic supplementary material.

Supplementary Material 1

## Data Availability

The datasets generated during and/or analyzed during the current study are available from the corresponding author upon reasonable request.

## Abbreviations

CK: Creatine Kinase
CK-MM: Creatine kinase type M
CK-BB: Creatine kinase type B
CK-MB: Creatine kinase type M and B
Mt-CK: Mitochondrial creatine kinase
Macro-CK: Macro creatine kinase
CRC: Colorectal cancer
OS: Overall survival
DFS: Disease-free survival
CEA: Carcinoembryonic antigen
CA: Carbohydrate antigen
ROC: Receiver-operating characteristic
AUC: Area under the curve
HR: Hazard ratio
CI: Confidence interval
AJCC: American Joint Committee on Cancer
