# Supplementary material for: Prognostic value of creatine kinase (CK)-MB to total-CK ratio in colorectal cancer patients after curative resection
Source: BMC Cancer. 2024 Apr 29;24:543. doi: 10.1186/s12885-024-12307-5 (PMC11059638; doi:10.1186/s12885-024-12307-5)
Supplement: Supplementary file 1 — Supplementary Material 1 [file 12885_2024_12307_MOESM1_ESM.docx]

**Supplementary Tables and Figures：**

| Years | Normal patients | Abnormal patients |
| --- | --- | --- |
|  | n=1177 | n=95 |
| 2017 | 134(11%) | 32(34%) |
| 2018 | 324(28%) | 16(17%) |
| 2019 | 381(32%) | 27(28%) |
| 2020 | 274(23%) | 17(18%) |
| 2021 | 64(5.4%) | 3(3.1%) |

**Supplementary Table 1. The number and proportion of patients included each year.** Data were shown as numbers (percentages).

| Follow-up time(month) | Total | Normal patients | Abnormal patients |
| --- | --- | --- | --- |
|  | n=1272 | n=1177 | n=95 |
| Median | 32.6 | 32.4 | 35.7 |
| 95%CI | 30.96-34.24 | 30.72-34.08 | 27.40-43.93 |

**Supplementary Table 2. The median follow-up time for the study population using the Reverse Kaplan-Meier method.** CI, confidence interval.

| Biomarkers |  |  | CEA | CA50 | CA19-9 | CA242 | CA72-4 |
| --- | --- | --- | --- | --- | --- | --- | --- |
| CK-MB/CK | Total | Rho | 0.215 | 0.156 | 0.154 | 0.168 | 0.158 |
|  |  | P | <0.001 | <0.001 | <0.001 | <0.001 | <0.001 |
|  | Stage I | Rho | 0.220 | 0.252 | 0.156 | 0.188 | 0.138 |
|  |  | P | 0.002 | 0.007 | 0.09 | 0.009 | 0.2 |
|  | Stage II | Rho | 0.198 | 0.043 | 0.116 | 0.083 | 0.046 |
|  |  | P | <0.001 | 0.5 | 0.05 | 0.08 | 0.4 |
|  | Stage III | Rho | 0.215 | 0.188 | 0.167 | 0.203 | 0.219 |
|  |  | P | <0.001 | <0.001 | <0.001 | <0.001 | <0.001 |
|  | Male | Rho | 0.205 | 0.138 | 0.152 | 0.188 | 0.101 |
|  |  | P | <0.001 | 0.003 | 0.001 | <0.001 | 0.03 |
|  | Female | Rho | 0.229 | 0.178 | 0.154 | 0.127 | 0.232 |
|  |  | P | <0.001 | 0.001 | 0.002 | 0.003 | <0.001 |
|  | Age(≤60) | Rho | 0.262 | 0.172 | 0.179 | 0.206 | 0.160 |
|  |  | P | <0.001 | <0.001 | <0.001 | <0.001 | 0.001 |
|  | Age(＞60) | Rho | 0.166 | 0.142 | 0.131 | 0.133 | 0.157 |
|  |  | P | <0.001 | 0.003 | 0.006 | <0.001 | 0.001 |

**Supplementary Table 3.** **The correlation analysis of CK-MB/CK and other biomarkers.** Rho and P values from Spearman's correlation test. CK-MB, creatine kinase type M and B; CK, creatine kinase.

| Variable | χ^2^ | P value |
| --- | --- | --- |
| CK-MB/CK | 0.037 | 0.8 |
| CA19-9 | 0.342 | 0.6 |
| CA242 | 0.561 | 0.5 |

**Supplementary Table 4. Evaluating the proportional hazards assumption of the Cox model by the Schoenfeld residual test.** χ^2^ and P values from Schoenfeld residual test.

**
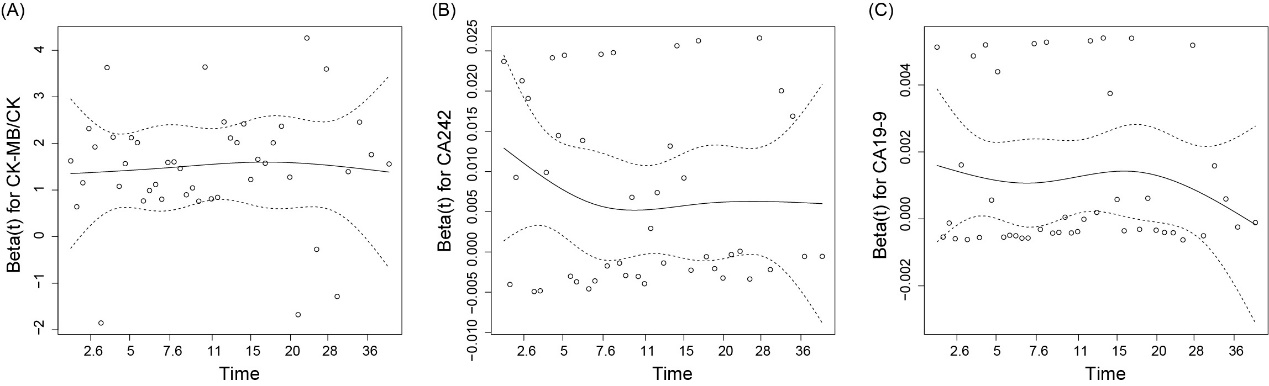
**

**Supplementary Figure 1. The residual plot for CK-MB/CK, CA242, and CA19-9.** CA, carbohydrate antigen; CK-MB, creatine kinase type M and B; CK, creatine kinase.


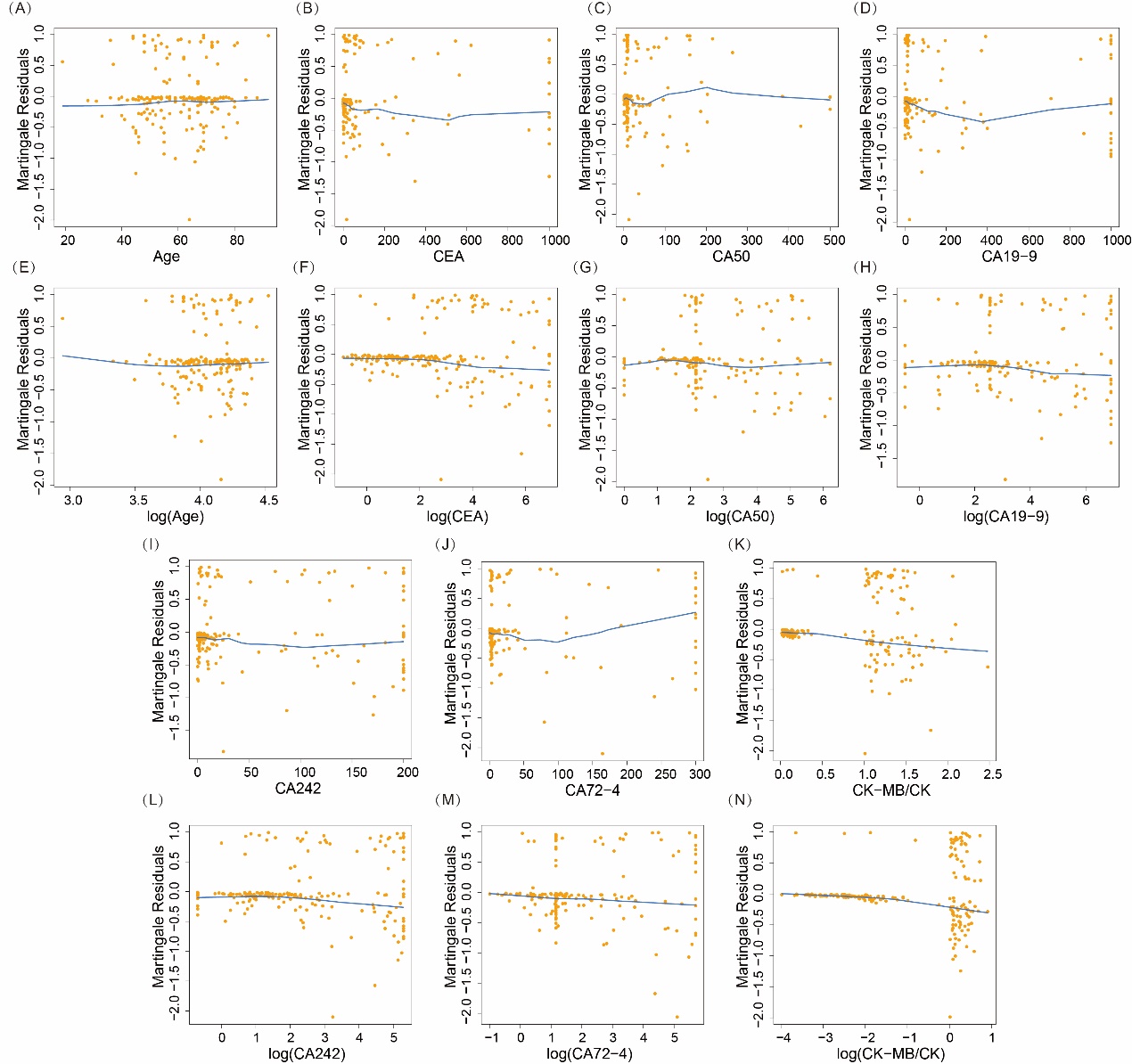


**Supplementary Figure 2**. **Martingale residual scatter plot of Cox regression analyses on overall survival.** Abbreviations: CEA, carcinoembryonic antigen; CA, carbohydrate antigen; CK-MB, creatine kinase type M and B; CK, creatine kinase.


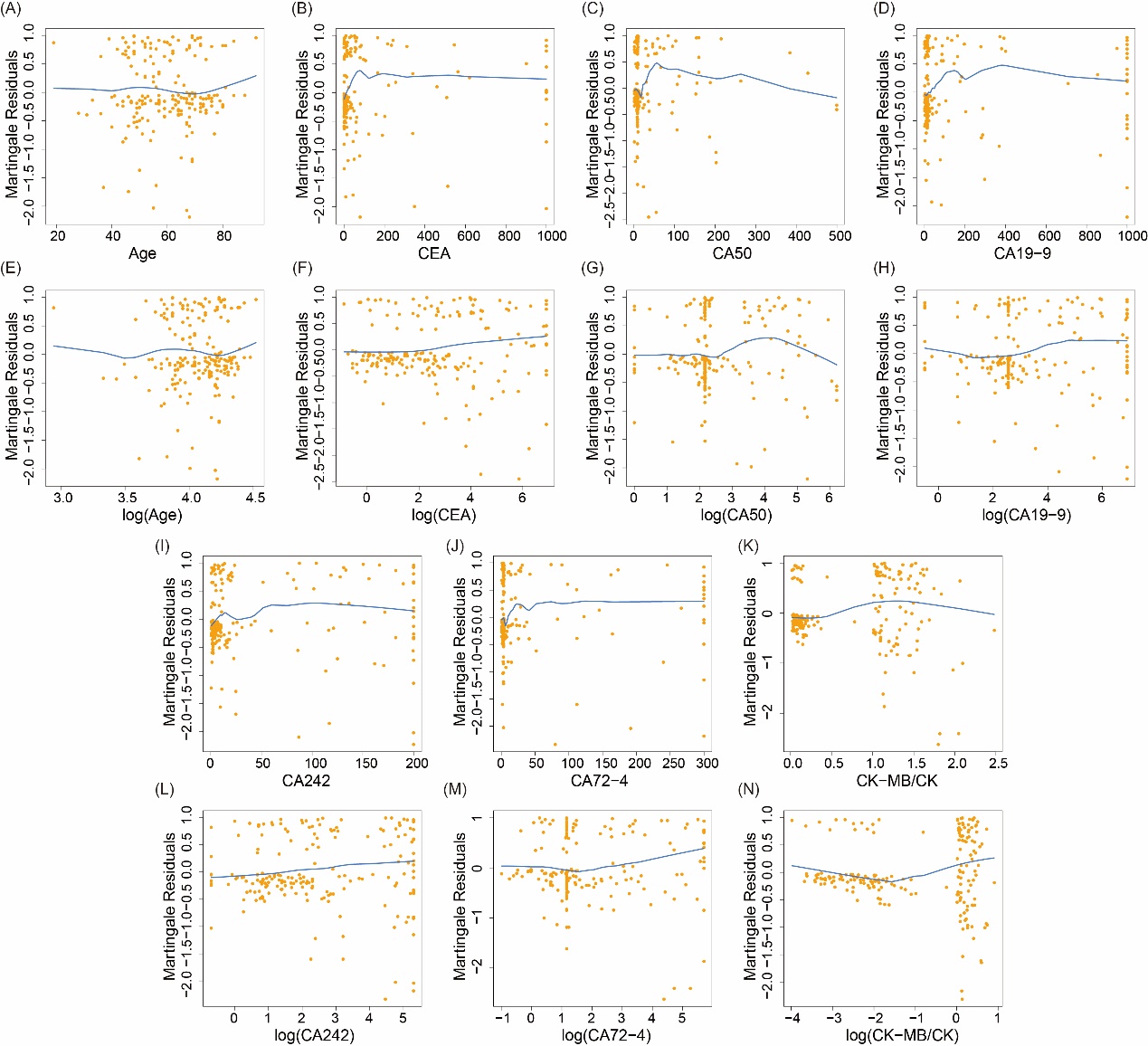


**Supplementary Figure 3**. **Martingale residual scatter plot of Cox regression analyses on disease-free survival.** Abbreviations: CEA, carcinoembryonic antigen; CA, carbohydrate antigen; CK-MB, creatine kinase type M and B; CK, creatine kinase.


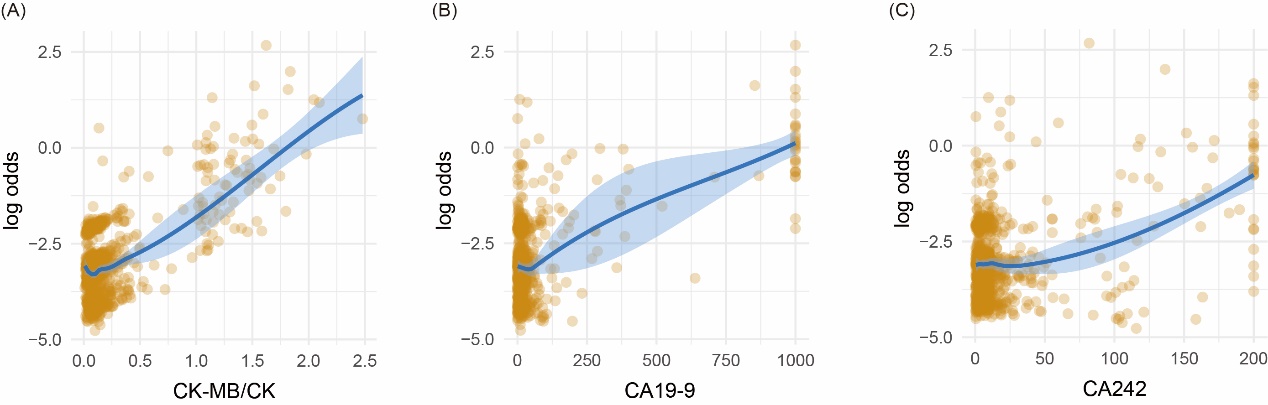


**Supplementary Figure 4**. **Scatter plot to verify linear conditions in logistic regression analysis.** Abbreviations: CA, carbohydrate antigen; CK-MB, creatine kinase type M and B; CK, creatine kinase.
